# Elevated plasma triglycerides and growth rate are early indicators of reproductive status in post-spawning female steelhead trout (*Oncorhynchus mykiss*)

**DOI:** 10.1093/conphys/coz038

**Published:** 2019-07-26

**Authors:** Laura E Jenkins, Andrew L Pierce, Neil D Graham, Lea R Medeiros, Douglas R Hatch, James J Nagler

**Affiliations:** 1 Department of Biological Sciences and Center for Reproductive Biology, University of Idaho, Moscow, ID, USA; 2 Fishery Science Department, Columbia River Inter-Tribal Fish Commission, Portland, OR, USA

**Keywords:** Estradiol-17*β*, growth, life history, reproduction, skip spawning, triglycerides

## Abstract

Many iteroparous fishes spawn after skipping one or more yearly cycles, which impacts recruitment estimates used for fisheries management and conservation. The physiological mechanisms underlying the development of consecutive and skip spawning life histories in fishes are not well understood. In salmonids, lipid energy reserves and/or growth are thought to regulate the initiation of reproductive maturation during a critical period ~1 year prior to spawning. The fasting spawning migration of summer-run steelhead trout (*Oncorhynchus mykiss*) results in significant depletion of energy reserves during the proposed critical period for repeat spawning. To determine whether and when lipid energy reserves and growth influence repeat spawning, measures of lipid energy reserves, growth rate and reproductive development were tracked in female steelhead trout from first to second spawning as a consecutive or skip spawner in captivity. Plasma triglyceride (TG) levels and growth rate were elevated by 10 weeks after spawning in reproductive (i.e. consecutive spawning) versus non-reproductive (i.e. skip spawning) individuals. Muscle lipid (ML) levels, condition factor and plasma estradiol levels increased at later time points. The early differences in plasma TG levels and increases in growth rate are attributable to differential rates of feeding and assimilation between the groups following spawning. A year after spawning, plasma TG levels, MLs and growth rate decreased in consecutive spawners, attributable to transfer of lipid reserves into the ovary. During the year prior to second spawning, energy reserves and plasma estradiol levels were higher in reproductive skip spawners versus consecutive spawners, reflecting the energy deficit after first spawning. These results suggest that the decision to initiate ovarian recrudescence occurs by 10 weeks after first spawning and are consistent with the differences in energy reserves acquired following spawning being a consequence of that decision. This information will increase the success of conservation projects reconditioning post-spawning summer-run steelhead trout.

## Teaser

Activity patterns of desert subterranean tuco-tucos were measured using light-loggers and accelerometers to understand interrelationships among environment, behavior and physiology. Underground temperatures attain minimal values around noon, favoring diurnal time on surface in winter. Timing of highest overall activity shifts to night-time upon transference from field to lab constant conditions.

## Introduction

Skipped spawning is common in seasonally breeding iteroparous fishes (Rideout and Tomkiewicz, 2011). After the first spawning event, individuals may spawn again after a 1-year interval (consecutive spawners) or after skipping 1 or more years (skip spawners). Many fish populations of conservation concern exhibit consecutive and skip spawning, which significantly impacts management, particularly of exploited stocks (Rideout *et al*., 2005). Reproductive schedules are phenotypically plastic and respond to environmental conditions (Thorpe *et al*., 1998; Rideout *et al*., 2005; Chaput and Benoit, 2012), suggesting that reproductive decisions and subsequent life history diversity will be sensitive to climate change, for example. Energetic status is thought to be the main determinant of the decision to engage in reproductive activity (i.e. initiate ovarian recrudescence) as a consecutive spawner or to defer reproductive activity for a future year leading to skip spawning (Thorpe, 1994; Rideout *et al*., 2005). Many of the fish species that exhibit skip spawning are capital breeders that fund reproduction from energy stores acquired prior to the majority of reproductive investment (McBride *et al*., 2015). This has resulted in the idea of a threshold level of energy reserves required to successfully complete gonadal development, spawning and associated activities such as migration. In this reaction norm framework, an individual’s condition or level of energy reserves interacts with a genetically determined threshold to generate a decision to either engage in reproductive activity or to remain reproductively inactive for the given reproductive cycle (Hutchings, 2011). However, the proximate physiological mechanisms involved in the decision to initiate or defer reproductive activity as a post-spawning adult are not fully understood.

The critical period hypothesis of salmonid maturation was developed for Atlantic salmon (*Salmo salar*) and proposes that the maturation decision takes place during a seasonally defined critical period ~1 year before spawning and that it is permissively gated by stores of metabolic fuels (Thorpe *et al*., 1998; Thorpe, 2007). The aspect of metabolic fuel storage that gates entry into a reproductive cycle, which is thought to occur at the transition to secondary oocyte growth in salmonids (Campbell *et al*., 2006; Lubzens *et al*., 2010), is not known but is functionally defined as either the absolute level or the rate of change of body size (growth) and/or lipid reserves (Thorpe, 2007; Taranger *et al*., 2010). Triglycerides (TGs) are the primary form in which lipids are stored for energy in fishes (Sheridan, 1994) and are a reasonable representation of lipid energy reserves. Reproductive decisions in salmonids have been most studied in the context of maturation (i.e. puberty). It is reasonable to assume that similar mechanisms operate in repeat spawning as in first time spawning, although energy depletion from the initial spawning event will also play a role. Early gametogenesis was found to be under energetic control during a critical period approximately a year before repeat spawning in winter flounder (*Pleuronectes americanus*) (Burton, 1994). Although considerable support for such critical periods exists, to our knowledge, the timing of the maturation decision window and the relative importance of lipid reserves and growth rate in the initiation of recrudescence have not been precisely delineated in any species.

The anadromous rainbow trout (*Oncorhynchus mykiss*, steelhead trout) provides a model species for studying the reproductive decisions underlying consecutive and skip spawning. Steelhead trout spawn in the spring in cold freshwater streams, the resulting juveniles migrate to the ocean to feed and grow and then they return to their natal stream to spawn (Burgner *et al*., 1992, Quinn, 2005). Steelhead trout display a diverse and phenotypically plastic suite of life histories, including freshwater resident and anadromous forms, variation in size and age at seaward migration, variation in size and age at initial maturation and consecutive and skip spawning (Nielsen *et al*., 2011; Courter *et al*., 2013; Moore *et al*., 2014). In coastal ‘winter-run’ populations steelhead trout return to freshwater with developed gonads shortly before spawning. However, in ‘summer-run’ populations, such as those in the interior Columbia River Basin (CRB), steelhead trout enter freshwater with immature gonads in late summer and complete gonadal development while fasting and migrating to spawning areas to spawn the following spring (Quinn *et al*., 2016). Summer-run steelhead trout are considered capital breeders, with the energetic demands of migration, gonadal development and spawning resulting in an extreme energy deficit and high post-spawning mortality (Penney and Moffitt, 2014b). In interior CRB summer-run populations, the incidence of skip spawning increased with migration distance (Keefer *et al*., 2008), consistent with regulation by energetic status.

Reconditioning of post-spawning steelhead trout (kelts) has been developed as a conservation measure for declining populations of interior summer-run CRB steelhead trout listed as threatened under the US Endangered Species Act (ESA) (Hatch *et al*., 2013; Trammell *et al*., 2016). Kelt reconditioning projects are being implemented at several locations in the interior CRB and aim to increase the productivity of steelhead trout populations by allowing wild-origin fish the opportunity to spawn again. Kelts are captured after spawning, held and fed in freshwater and then released to migrate upstream and spawn again. Kelts collected for reconditioning are predominantly female, and some projects only recondition female fish (Hatch *et al*., 2013; Hatch *et al*., 2016). The consecutive and skip spawning life histories are observed in reconditioned female kelts and vary significantly in proportion by location and year (Hatch *et al*., 2016; Pierce *et al*., 2017). Individuals determined to be reproductive after reconditioning over the summer are released to spawn as consecutive spawners, whereas fish determined to be non-reproductive after a summer of reconditioning must be held for an additional year until they can be released as reproductive skip spawners. This adds complexity to the management of kelt reconditioning projects. Thus, understanding the timing and basis of reproductive decisions in repeat spawning female steelhead trout will directly assist in the management of CRB steelhead kelt reconditioning projects. More generally, advances in understanding of the physiology of reproductive life history decisions in repeat spawning steelhead trout would be expected to lead to improvements in the management of other fish populations displaying consecutive and skip spawning.

To facilitate studies on steelhead trout kelt reconditioning, an experimental system was developed using non-ESA-listed hatchery-origin female summer-run steelhead trout returning to Dworshak National Fish Hatchery (DNFH) on the Clearwater River in Idaho, USA. The spawning migration for this population (nearly 800 km) approaches the maximum for steelhead trout. DNFH steelhead trout fast from freshwater entry in August to September through spawning in February to April resulting in extreme energy depletion. Fish returning to DNFH are captured and held in tanks enabling repeated sampling to observe their recovery and reproductive development. Additional advantages of this system are that these fish are of uniform genetic stock, origin and age, have uniform and known spawn timing and demonstrate both consecutive and skip spawning life histories (Jenkins *et al*., 2018). The objectives of this study were (i) to determine when the decision occurs to become reproductively active as a consecutive spawner, (ii) to determine whether and when growth rates and lipid reserves influence this decision and (iii) to assess how this decision is impacted by recovery from first spawning.

## Materials and methods

### Fish

Female steelhead trout *O. mykiss* were captured after returning on their first spawning migration and ascending the adult ladder trap at DNFH, on the Clearwater River in Ahsahka, ID, USA. Fish were collected for up to several weeks prior to spawning and maintained in holding ponds supplied with North Fork Clearwater River water. Females were selected for spawning based on a minimum criterion of 70 cm fork length (FL). Fish in good and fair condition with no visible wounds were selected for this study (Hatch *et al.*, 2013, 2016). Nearly all females included in the study became sexually mature for the first time at age 4; age was confirmed for a subset of the study fish (L. Jenkins et al., unpublished data).

### Spawning

In February to April 2015 and 2016, *n* = 150 and 164 females, respectively, were selected for this study. Fish were anesthetized using AQUI-S 20E (AquaTactics Inc., Kirkland, WA; 75 ml 1000 L^−1^ water) and manually ‘air spawned’. Air spawning consisted of inserting a 16-gauge pneumatic-hypodermic needle through the mid-body cavity wall just posterior to the pelvic fin, blowing 17.2–20.7 kpa oxygen into the body cavity and collecting eggs from the urogenital opening (Leitritz and Lewis, 1976). Fish were individually tagged using 12 mm passive integrated transponder tags (Biomark Inc., Boise, ID) inserted near the pelvic girdle.

### Sampling

At spawning and at 10-week intervals thereafter fish were sampled for FL (cm), wet mass (kg), muscle lipid (ML, %) level and blood. Wet mass at first spawning was taken after eggs were removed and was corrected for any residual eggs remaining in the body cavity as previously described (Jenkins *et al*., 2018), hereafter referred to as somatic mass. Subsequent measurements of body mass included any new ovarian growth. ML level was measured by microwave energy meter (Fish Fatmeter model 692, Distell Inc., West Lothian, UK) using the Trout-1 setting, as previously validated for rainbow trout (Caldwell *et al*., 2013). Blood (3 ml) was taken from the caudal vein using a heparinized (ammonium heparin, 10 mg ml^−1^, Sigma-Aldrich, St. Louis, MO) 20-gauge, 3.8 cm needle and syringe. Blood was centrifuged at 8300 G for 5 min. The plasma was removed, frozen on dry ice in the field, and then later stored at −80°C. Sampling continued at 10-week intervals until spawning occurred again ~1 year later (50 weeks) for consecutive spawning 2015 fish, until ~1 year plus 30 weeks after spawning for 2015 fish that skipped spawning or until 30 weeks after spawning for 2016 spawn year fish.

### Fish husbandry

After spawning fish were placed in 4.6 m diameter outdoor tanks, with a water height of 1.5 m located at DNFH. Tanks were supplied with water at a flow of ~200 L min ^− 1^ drawn from the North Fork Clearwater River, with a seasonally varying temperature profile (4.9–11.0°C). Fish were fed *ad libitum* a mixture of boiled krill (*Euphausia superba*, Atlantic Pacific Products Inc., Kingston, RI) and pellets (Biobrood 6 mm pellet size, BioOregon Inc., Longview, WA) top coated with menhaden oil (Argent Aquaculture LLC, Redmond, WA) and freeze-dried decapsulated *Artemia* cysts (American Brine Shrimp, Ogden, UT) for increased palatability. At spawning, fish were prophylactically treated for bacterial infection with oxytetracycline (Durvet, Blue Springs, Missouri; 20 mg kg^−1^ body mass) and for parasitic gill copepods (*Salmincola californiensis*) with emamectin (Sigma-Aldrich, St. Louis, Missouri; 200 μg kg^−1^ body mass), both via intraperitoneal injection. Oxytetracycline injections continued at 10-week intervals during sampling, with emamectin injections applied only when copepods were visible on the gills. Tanks were treated with formalin (Syndel USA, Portland, OR; flow through treatment, 1:6000 for 1 h daily) to control *Saprolegnia*.

### Survival

Mortality occurred during reconditioning as expected for steelhead trout kelts (Hatch *et al*., 2013). On average, 55% of the mortality occurred within 10 weeks of spawning (47% and 62% for 2015 and 2016, respectively). Survival to 30 weeks after spawning was 29% in 2015 (43/150 fish) and 18% in 2016 (30/163). Survival for fish that did not spawn 1 year after first spawning was 17% in 2015 (25/150) and 14% (21/150) to 1 year plus 30 weeks after first spawning. In November 2016, ~35 weeks after first spawning for the 2016 fish, 1 year and 35 weeks after first spawning for the 2015 fish and ~15 weeks prior to second spawning for the 2015 skip spawners and the 2016 consecutive spawners, all fish died due to an equipment malfunction. A necropsy was performed on all mortalities.

### Assays

Plasma estradiol-17*β* (E2) levels were measured by ELISA (Biosense, Cayman Chemical, Ann Arbor, MI). Steroids were extracted from plasma using ether extraction, re-suspended in assay buffer and assayed in triplicate. The intra- and inter-assay coefficient of variation was 8.0% and 7.1%, respectively. Plasma TG concentration was measured using a VetTest (Idexx, Westport, ME), as validated for use in *Oncorhynchus* spp. (Meador *et al*., 2006).

### Morphometric analysis

Fulton’s condition factor (K) was calculated as

$\mathrm{K}=100\ast \mathrm{body}\ \mathrm{mass}\ (\mathrm{g})\ast {(\mathrm{fork}\ \mathrm{length}\ (\mathrm{cm}))}^{-3}$.

Mass specific growth rate (MSGR) was calculated as:

$\%\
\mathrm{body}\ \mathrm{mass}\ \mathrm{gain}^{\ast} {\mathrm{day}}^{-1}=100
^{\ast} (\ln (\mathrm{body}\ \mathrm{mass}\ \mathrm{final})\hbox{--} \ln\\ (\mathrm{body}\ \mathrm{mass}\ \mathrm{initial})){\mathrm{day}\mathrm{s}}^{-1}$.

Length specific growth rate (LSGR) was calculated in the same manner as that for mass.

### Determination of reproductive status

Reproductive status was assigned in early autumn, 30 weeks after spawning, based on complete separation of fish into two E2 concentration groups (high levels = reproductive, low levels = non-reproductive) and confirmed by spawning of survivors (Jenkins *et al*., 2018) or at necropsy for pre-spawn mortalities by examining developing ovaries for large oocytes.

In Year 1 following first spawning, consecutive spawners were reproductive and skip spawners were non-reproductive. In the year prior to second spawning, both consecutive and skip spawners were reproductive. Reproductive skip spawners had to survive Year 1 as non-reproductive and be assigned as reproductive in Year 2.

### Statistical analysis

Fish were first compared based on reproductive status in Year 1. Reproductive (*n* = 13, 2015; *n* = 12, 2016) and non-reproductive fish (*n* = 30, 2015; *n* = 18, 2016) were compared at 10-week intervals in a time series starting at spawning. Fish of the 2015 spawn year were then compared based on reproductive interval: consecutive spawners (Year 1, *n* = 13) and reproductive skip spawners (Year 2, *n* = 18) were compared at the same relative time points during the year prior to second spawning.

Two-way repeated measures analysis of variance (ANOVA) was employed to test for time, group, interaction and subject effects on TG levels, ML levels, K, MSGR, LSGR and E2 levels. E2 levels were log_10_-transformed, and ML levels were arcsine square root transformed prior to analysis to conform to assumptions of normality. Where significant effects were found, one-way repeated measures ANOVA was used to assess the effects of time and reproductive status or interval on individual fish, followed by Tukey’s honestly significant difference (HSD) test or a *T*-test. *T*-tests were used to assess differences in individual trajectories for reproductive and non-reproductive fish from 0 to 10 weeks (TG) and from 10 to 20 weeks (E2) in the year following first spawning, assessed as a percentage of the level at the previous time period.

Of the 2015 consecutive spawners (*n* = 13) in 2015, *n* = 12 were included in the analysis during the year following first spawning, as one fish was excluded due to a missing sampling point as required by repeated measures ANOVA. Of the 2015 non-reproductive skip spawners (*n* = 30), *n* = 20 were analysed, as nine fish had missing sampling points and one fish was excluded due to a distinctly non-representative negative TG, growth rate, ML and K trajectory starting in early summer (20 weeks post-spawn), followed by death 1 year after spawning. Of the 2016 consecutive spawners (*n* = 12), *n* = 10 consecutive spawners and *n* = 18 non-reproductive skip spawners were analysed, as two consecutive spawners had missing sampling points. Additional individuals were excluded from the TG analysis due to missing sampling points (one consecutive spawner 2015, one non-reproductive skip spawner 2016 and five reproductive skip spawners 2015). The Rout Outlier Test was used to detect and remove outliers (0.3% average number of outliers per group). Unless otherwise indicated, all statistical analyses were conducted with PRISM software version 7.0 (GraphPad Inc., La Jolla, CA). Results are reported as significant when *P* < 0.05.

**Table 1:** Two-way repeated measures ANOVA test statistics for each dependent variable tracked over the year after first spawning (2015, 2016) for reproductive and non-reproductive groups of female steelhead trout

Measure	Source of variation	2015		2016	
		*F* (DF_n_, DF_d_)	*P*-value	*F* (DF_n_, DF_d_)	*P*-value
TG	Time	*F* (4.2, 122.8) = 33.5	*P* < 0.001	*F* (2.3, 57.4) = 14.7	*P* < 0.0001
	Group	*F* (1, 29) = 10.08	*P* = 0.0035	*F* (1, 25) = 8.275	*P* = 0.0081
	Interaction	*F* (5, 145) = 8.783	*P* < 0.001	*F* (3, 75) = 2.286	***P* = 0.0856**
	Subject	*F* (29, 145) = 2.923	*P* < 0.001	*F* (25, 75) = 1.837	*P* = 0.0232
ML	Time	*F* (2.3, 67.7) = 97.9	*P* < 0.001	*F* (1.3, 34.9) = 71.6	*P* < 0.0001
	Group	*F* (1, 30) = 1.652	***P* = 0.2085**	*F* (1, 26) = 6.446	*P* = 0.0174
	Interaction	*F* (5, 150) = 26.65	*P* < 0.001	*F* (3, 78) = 5.307	*P* = 0.0022
	Subject	*F* (30, 150) = 5.127	*P* < 0.001	*F* (26, 78) = 2.248	*P* = 0.0033
K	Time	*F* (2.2, 64.9) = 127.6	*P* < 0.001	*F* (1.5, 39.2) = 78.6	*P* < 0.0001
	Group	*F* (1, 30) = 5.480	*P* = 0.0261	*F* (1, 26) = 10.46	*P* = 0.0033
	Interaction	*F* (5, 150) = 7.868	*P* < 0.001	*F* (3, 78) = 9.740	*P* < 0.0001
	Subject	*F* (30, 150) = 10.09	*P* < 0.001	*F* (26, 78) = 3.669	*P* < 0.0001
MSGR	Time	*F* (3.3, 96.9) = 42.9	*P* < 0.0001	*F* (1.9, 47.3) = 42.5	*P* < 0.0001
	Group	*F* (1, 29) = 16.33	*P* = 0.0004	*F* (1, 25) = 10.10	*P* = 0.0039
	Interaction	*F* (4, 116) = 1.786	***P* = 0.1364**	*F* (2, 50) = 0.07	***P* = 0.9351**
	Subject	*F* (29, 116) = 1.358	***P* = 0.1294**	*F* (25, 50) = 1.7	***P* = 0.0554**
LSGR	Time	*F* (3.4, 93.9) = 45.31	*P* < 0.0001	*F* (1.7, 43.4) = 72.8	*P* < 0.0001
	Group	*F* (1, 28) = 4.908	*P* = 0.0350	*F* (1, 26) = 2.895	***P* = 0.1008**
	Interaction	*F* (4, 112) = 9.267	*P* < 0.0001	*F* (2, 52) = 1.909	***P* = 0.1584**
	Subject	*F* (28, 112) = 1.329	***P* = 0.1505**	*F* (26, 52) = 1.402	***P* = 0.1486**
E2	Time	*F* (2.8, 78.0) = 49.7	*P* < 0.0001	*F* (2.1, 51.8) = 22.8	*P* < 0.0001
	Group	*F* (1, 28) = 208.2	*P* < 0.0001	*F* (1, 25) = 105.4	*P* < 0.0001
	Interaction	*F* (5, 140) = 51.77	*P* < 0.0001	*F* (3, 75) = 40.46	*P* < 0.0001
	Subject	*F* (28, 140) = 2.096	*P* = 0.0027	*F* (25, 75) = 1.257	***P* = 0.2223**

Bolded *P*-values indicate non-significance.

### Ethics

Fish care and sampling were conducted in accordance with a protocol reviewed and approved by the University of Idaho Animal Care and Use Committee.

## Results

### Post-spawning reproductive status

Of fish that survived to 30 weeks after first spawning, 30% (13/43) and 40% (12/30) became reproductively active as consecutive spawners in 2015 and 2016, respectively. Of the 30 non-reproductive skip spawners from 2015, 70% survived to 1 year plus 30 weeks after spawning (21/30), and 86% became reproductively active skip spawners in 2016 (18/21). No evidence of arrested reproductive development after 20 weeks post-spawning (i.e. premature decreases in plasma E2 level) was detected in any individual.

### Time course following first spawning

Two-way repeated measures ANOVA found significant effects of group, time, group*time interactions and subject (Table 1).

**Figure 1:**
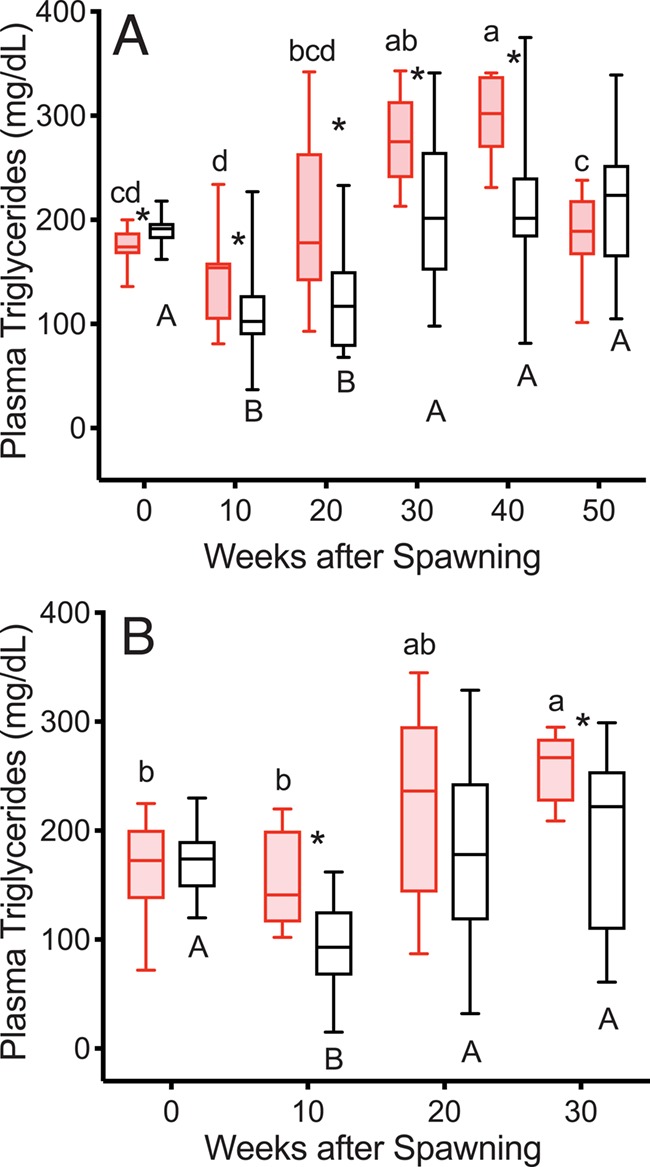
Plasma TG concentrations in female steelhead trout from the Clearwater River, Idaho, sampled in 2015 (A) and 2016 (B) at 10-week intervals following spawning such that analysis included reproductive (red, shaded boxes, *n* = 11, 10; 2015 and 2016, respectively) and non-reproductive fish (black, *n* = 20, 17; 2015 and 2016, respectively), box heights indicate interquartile range, horizontal lines within indicate the median and whiskers show the data range, time points within a group sharing the same letter do not differ significantly (repeated measures one-way ANOVA followed by Tukey’s HSD Test, *P* < 0.05), and asterisks indicate significant differences between groups at each time point (*T*-test, *P* < 0.05).

TG levels were greater in reproductive than in non-reproductive fish at 10 weeks after first spawning in both years, remaining that way except for Week 20 in 2016 and Week 50 in 2015 (Fig. 1). At the individual level, from Week 0 to Week 10, TG decreased in non-reproductive fish (19/20, 15/16 decreased; to 56% and 58% of Week 0 in 2015 and 2016, respectively) and stayed the same or decreased to a significantly lesser extent in reproductive fish (8/11, 5/10 decreased; to 84%, 100% of Week 0 in 2015 and 2016, respectively; *T*-test, *P* = 0.0102, 0.0036 in 2015 and 2016, respectively). After the 10-week time point, TG in non-reproductive fish returned to first-spawning levels at 20 (2016) or 30 (2015) weeks and for all following time points. TG increased over spawning levels in all reproductive fish by 30 weeks in both years. In 2015, TG levels were slightly but significantly greater in non-reproductive than in reproductive fish at the time of first spawning.

**Figure 2:**
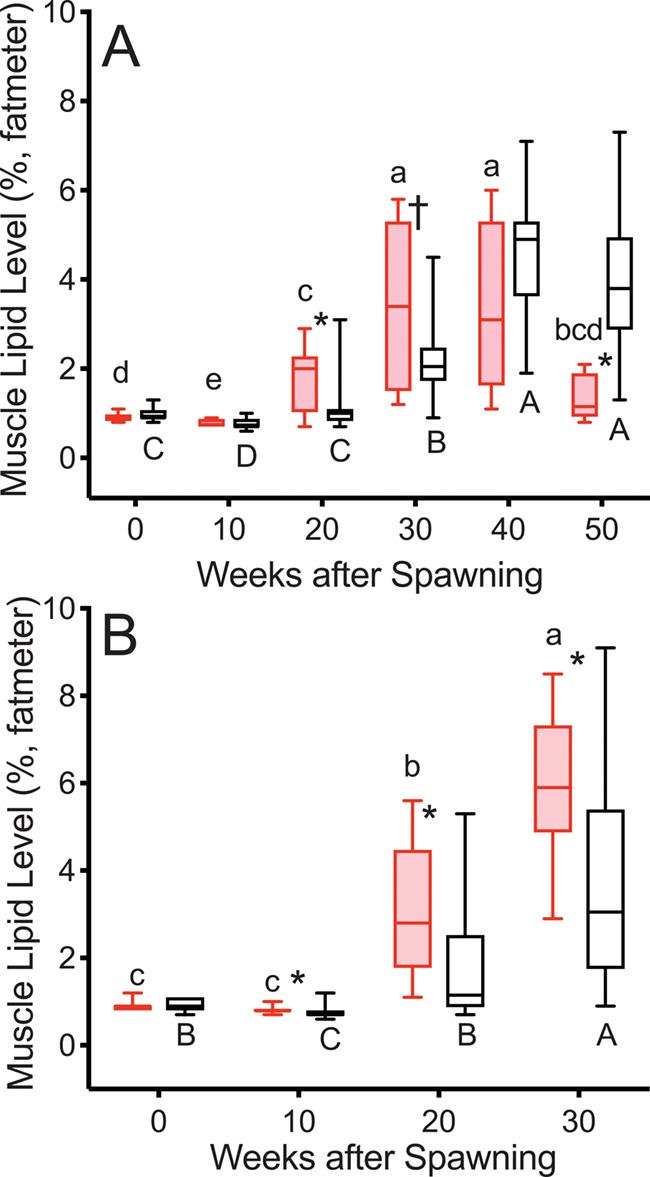
ML levels in female steelhead trout from the Clearwater River, Idaho, sampled in 2015 (A) and 2016 (B) at 10-week intervals following spawning, such that groups, box and whisker plots and significance indication are as in Fig. 1, and analysis included reproductive (*n* = 12, 10; 2015 and 2016, respectively) and non-reproductive fish (*n* = 20, 18; 2015 and 2016, respectively).

ML level was greater in reproductive than non-reproductive fish at Weeks 20 and 30 in 2015 and 2016 (Fig. 2). At Week 50 in 2015 ML level was greater in non-reproductive than in reproductive fish. ML level increased progressively from Week 10 to 30 in both groups and years.

**Figure 3:**
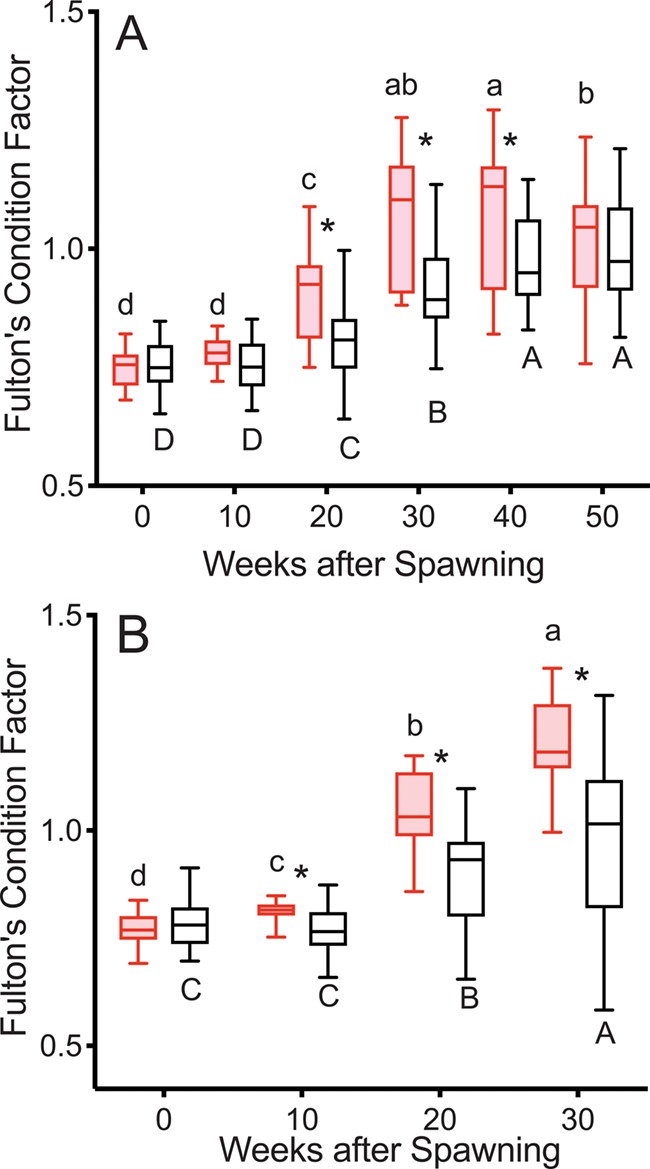
Fulton’s condition factor in female steelhead trout from the Clearwater River, Idaho, sampled in 2015 (A) and 2016 (B) at 10-week intervals following spawning, such that groups, box and whisker plots, significance indication and statistical analyses are as in Fig. 1, and analysis included reproductive (*n* = 12, 10; 2015 and 2016, respectively) and non-reproductive fish (*n* = 20, 18; 2015 and 2016, respectively).

K was greater in reproductive than non-reproductive fish at Weeks 20–40 in 2015 and at Weeks 10–30 in 2016 (Fig. 3). K increased progressively from Week 10 to Week 30 in reproductive fish in both years. K increased progressively from 10 to 40 weeks (2015) and 10 to 30 weeks (2016) in non-reproductive fish.

**Figure 4:**
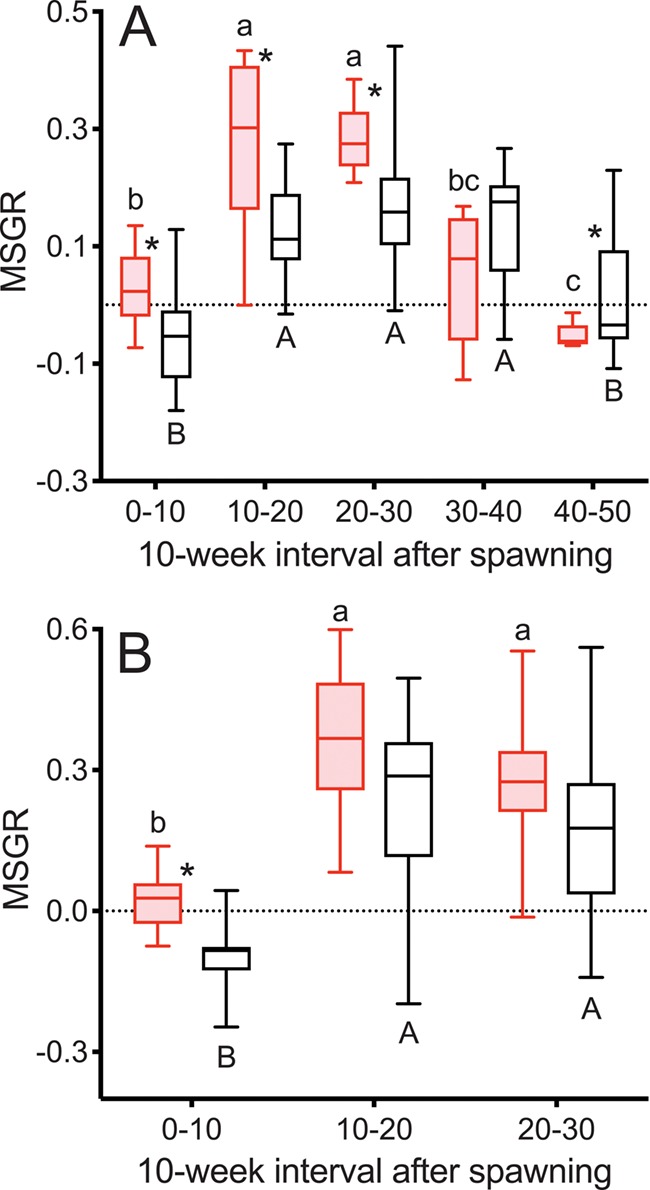
MSGR as % change in body weight per day in female steelhead trout from the Clearwater River, Idaho, sampled in 2015 (A) and 2016 (B) calculated over 10-week intervals following spawning, such that groups, box and whisker plots, significance indication and statistical analyses are as in Fig. 1, and analysis included reproductive (*n* = 10, 10; 2015 and 2016, respectively) and non-reproductive fish (*n* = 20, 17; 2015 and 2016, respectively).

MSGR was greater in reproductive than non-reproductive fish during Weeks 0–10 after first spawning in both years (Fig. 4), continuing for Weeks 10–20 and Weeks 20–30 in 2015. MSGR was positive for reproductive and negative for non-reproductive fish during Weeks 0–10 in both years (Fig. 4). MSGR increased strongly from Weeks 0–10 to Weeks 10–20 in both reproductive and non-reproductive fish in both years and remained high through Weeks 20–30. In 2015, MSGR declined from Weeks 20–30 to Weeks 30–40 in reproductive fish, reaching levels below that of Weeks 0–10 during Weeks 40–50. MSGR also declined from Weeks 30–40 to Weeks 40–50 in non-reproductive fish, returning to levels similar to Weeks 0–10. MSGR was greater in non-reproductive fish than reproductive fish over Weeks 40–50 in 2015.

**Figure 5:**
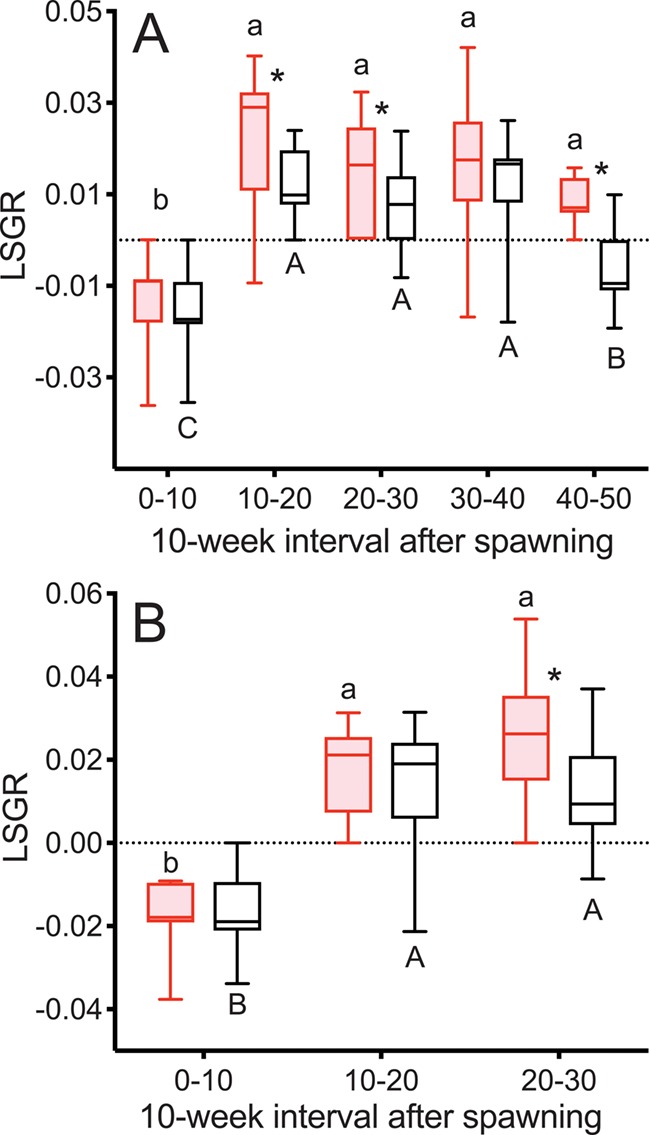
LSGR as % change in FL per day in female steelhead trout from the Clearwater River, Idaho, sampled in 2015 (A) and 2016 (B) at 10-week intervals following spawning, such that groups, box and whisker plots, significance indication and statistical analyses are as in Fig. 1, and analysis included reproductive (*n* = 11, 10; 2015 and 2016, respectively) and non-reproductive fish (*n* = 20, 18; 2015 and 2016, respectively).

LSGR was negative during Weeks 0–10 and increased during Weeks 10–20 in both reproductive and non-reproductive fish in both years (Fig. 5). LSGR subsequently decreased from Weeks 30–40 to 40–50 in non-reproductive fish in 2015. LSGR was greater in reproductive than non-reproductive fish at Weeks 10–20, 20–30 and 40–50 in 2015 and Weeks 20–30 in 2016.

**Figure 6:**
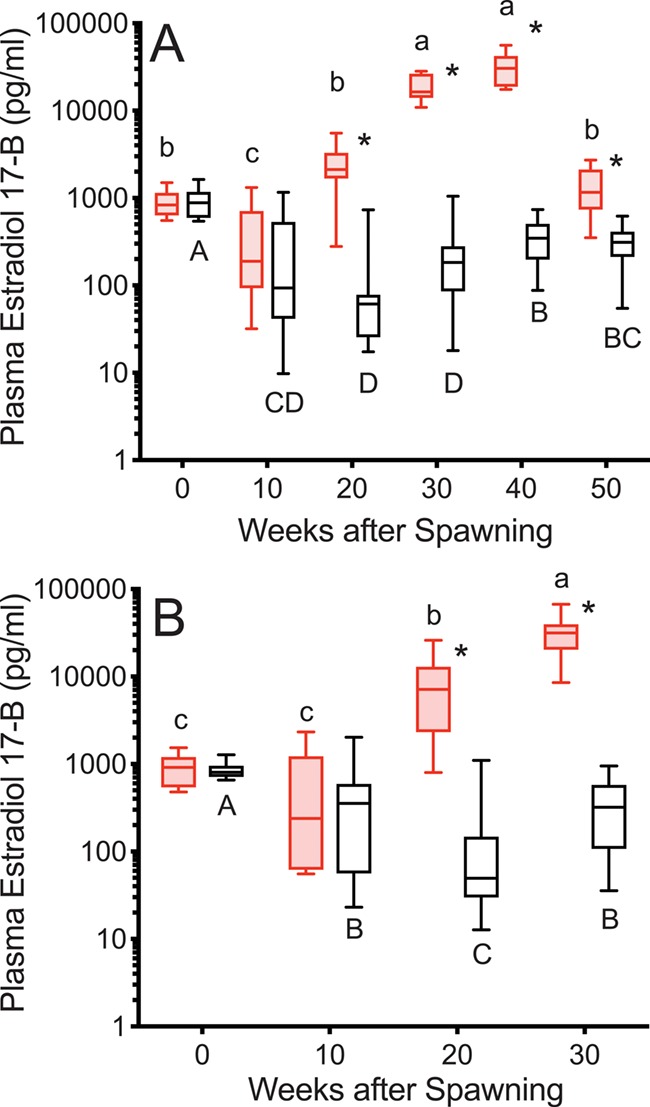
Plasma estradiol-17β concentrations in female steelhead trout from the Clearwater River, Idaho, sampled at 10-week intervals following first spawning in 2015 (A) and 2016 (B), such that groups, box and whisker plots, significance indication and statistical analyses are as in Fig. 1, and analysis included reproductive (*n* = 11, 10; 2015 and 2016, respectively) and non-reproductive fish (*n* = 20, 17; 2015 and 2016, respectively).

E2 levels were greater in reproductive than non-reproductive fish starting at 20 weeks after first spawning, remaining that way for the study duration in both years (Fig. 6). E2 levels decreased from spawning to Week 10 in both groups and years, except in the 2016 reproductive group (*P* = 0.1354). At the individual level, in reproductive fish in both years, log E2 levels increased from Week 10 to Week 20 (9/11, 10/10 increased; to 145%, 163% of Week 10 in 2015 and 2016, respectively) and decreased in non-reproductive fish (12/19, 15/17 decreased; to 97%, 81% of Week 10 in 2015 and 2016, respectively). Individual level changes were significantly different between groups (*T*-test, *P* = 0.0029, *P* < 0.0001 for 2015 and 2016, respectively). E2 levels increased again from Week 20 to Week 30 in reproductive fish, then decreased from Week 40 to Week 50 to levels similar to Week 0 in 2015. In non-reproductive fish in both years, E2 remained below first spawning levels, despite increasing from Weeks 20–30 (30–40 in 2015).

### Time course prior to second spawning

Two-way repeated measures ANOVA found significant effects of maturation group, time, group*time interactions and subject (Table 2).

**Table 2:** Two-way repeated measures ANOVA test statistics for each dependent variable in female steelhead trout tracked over the year prior to second spawning for consecutive spawners and reproductive skip spawners first spawned in 2015.

Measure	Source of variation	*F* (DF_n_, DF_d_)	*P*-value
TG	Time	*F* (2.3, 51.0) = 19.28	*P* < 0.0001
	Group	*F* (1, 22) = 23.74	*P* < 0.0001
	Interaction	*F* (3, 66) = 8.519	*P* < 0.0001
	Subject	*F* (22, 66) = 1.528	***P* = 0.0954**
ML	Time	*F* (1.3, 38.9) = 66.37	*P* < 0.0001
	Group	*F* (1, 29) = 120.7	*P* < 0.0001
	Interaction	*F* (3, 87) = 9.114	*P* < 0.0001
	Subject	*F* (29, 87) = 6.639	*P* < 0.0001
K	Time	*F* (1.5, 45) = 131.1	*P* < 0.0001
	Group	*F* (1, 29) = 54.40	*P* < 0.0001
	Interaction	*F* (3, 87) = 12.16	*P* < 0.0001
	Subject	*F* (29, 87) = 12.62	*P* < 0.0001
MSGR	Time	*F* (2.0, 54) = 33.12	*P* < 0.0001
	Group	*F* (1, 27) = 11.95	*P* = 0.0018
	Interaction	*F* (2, 54) = 23.30	*P* < 0.0001
	Subject	*F* (27, 54) = 1.361	***P* = 0.1658**
LSGR	Time	*F* (2.0, 58) = 42.72	*P* < 0.0001
	Group	*F* (1, 29) = 12.76	*P* = 0.0013
	Interaction	*F* (2, 58) = 12.31	*P* < 0.0001
	Subject	*F* (29, 58) = 1.642	***P* = 0.0543**
E2	Time	*F* (2.5, 71) = 229.6	*P* < 0.0001
	Group	*F* (1, 28) = 39.62	*P* < 0.0001
	Interaction	*F* (3, 84) = 31.18	*P* < 0.0001
	Subject	*F* (28, 84) = 1.342	***P* = 0.1536**

Bolded *P*-values indicate non-significance.

**Figure 7:**
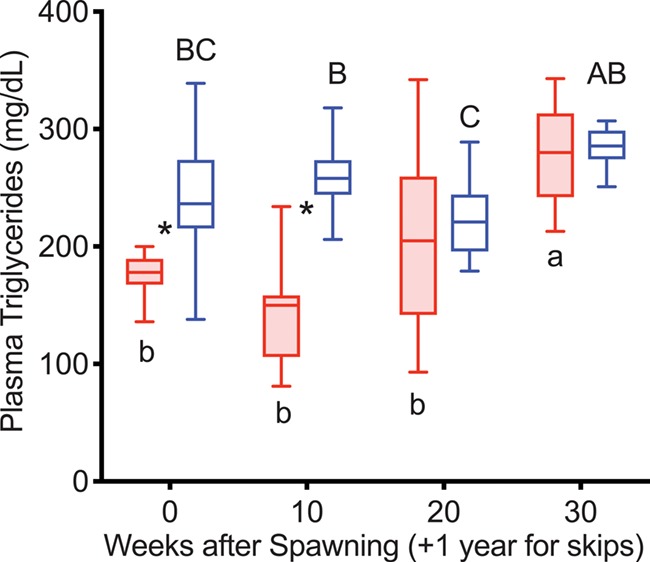
Plasma TG concentrations tracked over the year prior to second spawning in female steelhead trout from the Clearwater River, Idaho, where fish first spawned in 2015 were sampled at 10-week intervals in 2015 or 2016, and analysis included consecutive spawners (*n* = 12, red, shaded boxes) and reproductive skip spawners (*n* = 12, blue).

TG levels were greater in reproductive skip spawners than in consecutive spawners at Weeks 0 and 10 in the year prior to second spawning (Fig. 7). TG levels decreased in reproductive skip spawners from Week 10 to Week 20 and then increased in both groups from Week 20 to Week 30.

**Figure 8:**
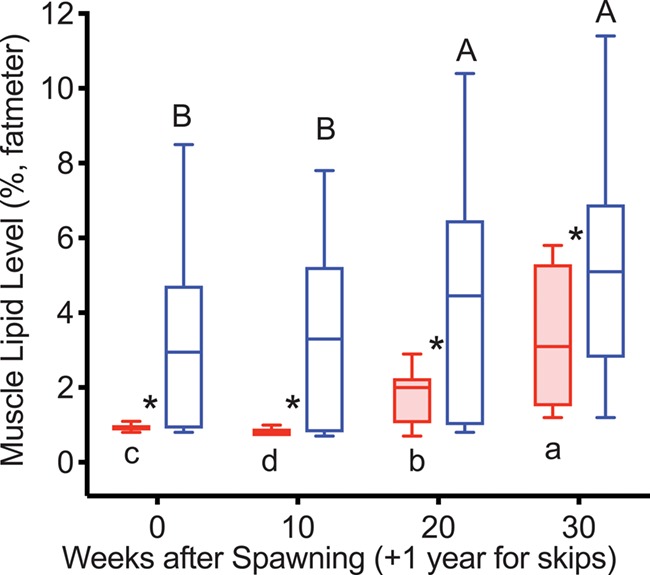
ML levels tracked over the year prior to second spawning in female steelhead trout from the Clearwater River, Idaho, such that groups are as in Fig. 7, box and whisker plots and significance indication are as in Fig. 1, and analysis included consecutive spawners (*n* = 13) and reproductive skip spawners (*n* = 18).

ML levels were greater in reproductive skip spawners than in consecutive spawners at all time points (Fig. 8). ML levels increased at Week 20 in both groups.

**Figure 9:**
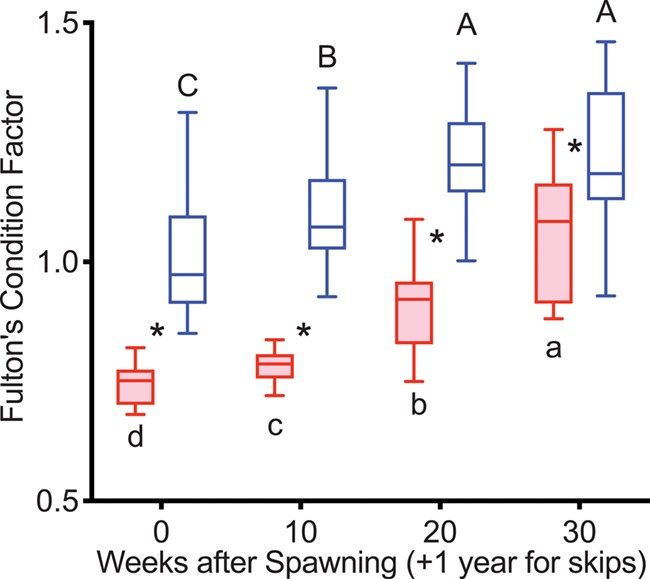
Fulton’s condition factor tracked over the year prior to second spawning in female steelhead trout from the Clearwater River, Idaho, such that groups are as in Fig. 7, box and whisker plots, significance indication and statistical analyses are as in Fig. 1, and analysis included consecutive spawners (*n* = 13) and reproductive skip spawners (*n* = 18).

K was greater in reproductive skip spawners than in consecutive spawners at all time points (Fig. 9). K increased progressively in consecutive spawners from Week 10 to 30 and in reproductive skip spawners from Week 0 to 20.

**Figure 10:**
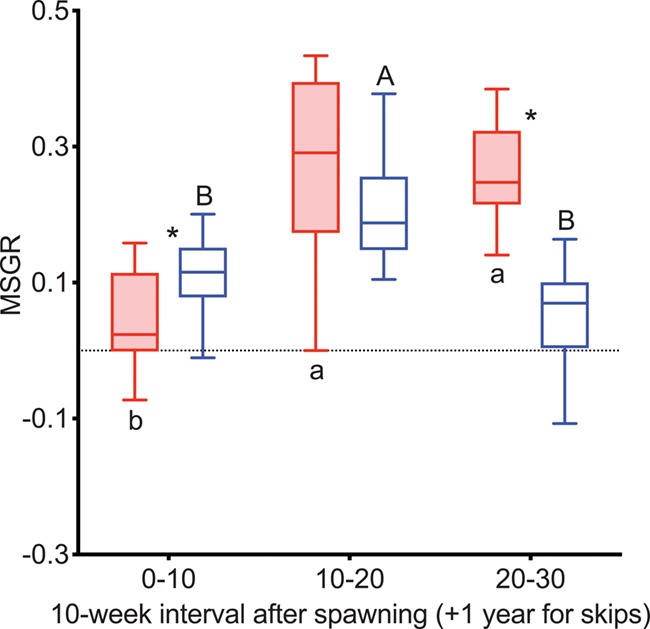
MSGR as % change in body weight per day tracked over the year prior to second spawning in female steelhead trout from the Clearwater River, Idaho, such that groups are as in Fig. 7, box and whisker plots, significance indication and statistical analyses are as in Fig. 1 and analysis includes consecutive spawners (*n* = 12) and reproductive skip spawners (*n* = 17).

MSGR was greater in reproductive skip spawners during Weeks 0–10, but greater in consecutive spawners during Weeks 20–30 (Fig. 10). MSGR increased from Weeks 0–10 to Weeks 10–20 in both groups. During Weeks 20–30 MSGR in reproductive skip spawners decreased to levels similar to Weeks 0–10.

**Figure 11:**
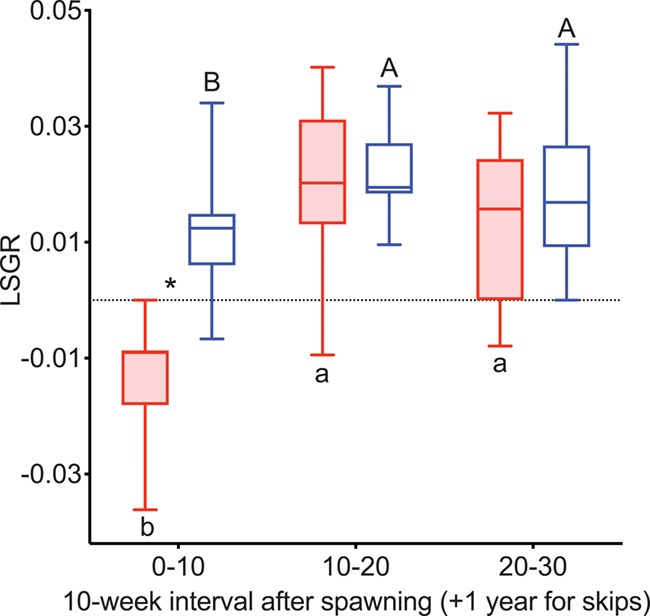
LSGR as % change in FL per day tracked over the year prior to second spawning in female steelhead trout from the Clearwater River, Idaho. Groups are as in Fig. 7, such that box and whisker plots, significance indication and statistical analyses are as in Fig. 1, and analysis included consecutive spawners (*n* = 13) and reproductive skip spawners (*n* = 18).

LSGR was greater in reproductive skip spawners than consecutive spawners (which had negative LSGR) during Weeks 0–10 (Fig. 11). LSGR increased from Weeks 0–10 to Weeks 10–20 in both groups.

**Figure 12:**
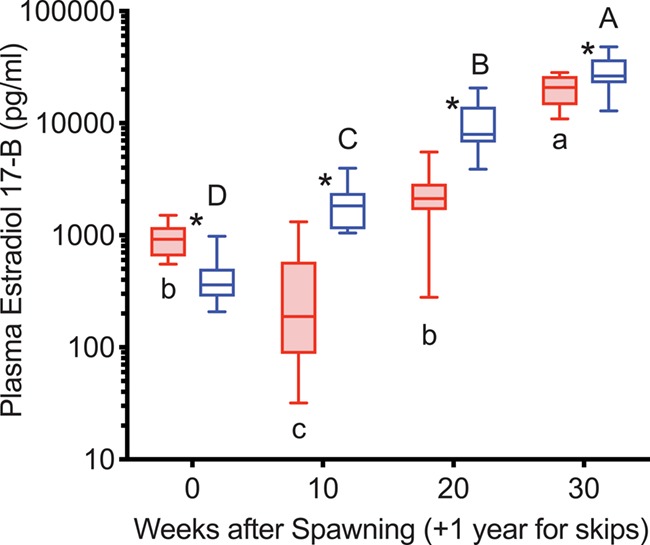
Plasma estradiol-17β concentrations tracked over the year prior to repeat spawning in female steelhead trout from the Clearwater River, Idaho, such that groups are as in Fig. 7, box and whisker plots, significance indication and statistical analyses are as in Fig. 1, and analysis includes consecutive spawners (*n* = 12) and reproductive skip spawners (*n* = 18).

E2 levels were greater in consecutive spawners than reproductive skip spawners at Week 0, but greater in reproductive skip spawners from 10 to 30 weeks (Fig. 12). From 0 to 10 weeks E2 levels increased in reproductive skip spawners and decreased in consecutive spawners. Thereafter, E2 levels increased for both groups.

## Discussion

The sequence of events over the year after first spawning tracked in this study (Figs 1–6) illustrates the timing of reproductive decisions and differences in energy acquisition and allocation between consecutive and skip spawning female steelhead trout. TG levels and growth rate in mass were significantly greater 10 weeks after first spawning in reproductive versus non-reproductive fish. This implies greater feeding in reproductive fish over this time period and is consistent with the decision to enter the next reproductive cycle having occurred by 10 weeks after first spawning. Reproductive fish accumulated greater energy reserves and grew faster than non-reproductive fish over the summer growing season in the year following first spawning, consistent with the decision to enter the next reproductive cycle having stimulated feeding, without precluding the opposite scenario. The sequence of events over the year prior to repeat spawning (Figs 7–12) illustrates the effect of recovery from first spawning on energy reserves, growth and reproductive decisions. The increase in E2 occurred at relatively earlier time points in the year prior to second spawning in reproductively active skip spawners than in fish recrudescing in the year immediately following first spawning (consecutive spawners), suggesting that in consecutive spawners reproductive development was delayed by the energetic or physiological demands of first spawning. Reproductive skip spawners had substantially greater energy reserves (i.e. ML and K) and E2 levels during oogenesis for second spawning. This likely allowed for greater reproductive investment in reproductive skip spawners versus consecutive spawners at the time of second spawning, as was found in our companion study (Jenkins *et al*., 2018). This study provides the first mechanistic look at the timing and physiological factors involved in reproductive decisions in repeat spawning female steelhead trout. These results will directly inform the management of kelt reconditioning conservation programs and advance knowledge about the underlying physiology of consecutive and skip spawning, an important issue in the management of many fish populations.

### Energy reserves

Energy reserves were assessed using three metrics, focusing on lipids as these have been proposed as particularly important in salmonid maturation (Thorpe *et al*., 1998): TG representing short-term lipid energy availability, ML representing long-term lipid energy stores and K, a measure of body shape used as a proxy for whole body energy stores in fishes (Sutton *et al*., 2000; Hanson *et al*., 2010). Energy reserves generally increased more rapidly during recovery from spawning in consecutive spawners but attained higher levels during the extended reconditioning period in skip spawners.

#### Year after first spawning

After spawning, TG levels were greater in reproductive fish from 10 to 40 weeks, with minor variation in significance between years and then decreased immediately before spawning. The divergence in circulating TG levels at 10 weeks was due to a significantly lesser decrease from Week 0 in individual fish reproductive in the year following first spawning. This is most likely because of greater food intake, assimilation and more rapid somatic recovery in reproductively active fish. However, we cannot exclude the possibility that lipid metabolism differed between reproductive and non-reproductive female steelhead trout during this period. Our interpretation that the increase in TG in reproductive fish was due to greater food intake is supported by the MSGR results discussed below. If our interpretation is correct, then physiological differences manifesting in the form of greater feeding motivation occurred between reproductive and non-reproductive fish by 10 weeks after spawning, implying that the decision to enter a reproductive cycle is linked to the difference in feeding motivation. This suggests two possible mechanisms: (i) the decision occurs late during the first 10 weeks or afterward, with increased feeding early on after spawning causing improved energetic status that leads to the initiation of ovarian recrudescence or (ii) the decision occurs prior to or early during the first 10 weeks, with the initiation of ovarian recrudescence stimulating increased feeding and nutrient assimilation. The second possibility is supported in part by a study in winter flounder, which linked recrudescence to high condition in the period immediately following first spawning, regardless of access to food during that period (Burton, 1994). Further, studies in Atlantic salmon and rainbow trout showed increased feed intake, growth and plasma levels of insulin-like growth factor-1 in maturing fish during early stages of maturation ~1 year before spawning (Kadri *et al*., 1996; Stead *et al*., 1999; Wilkinson *et al*., 2010). In addition, the presence of small ovarian follicles in rainbow trout and steelhead trout kelt ovaries immediately after ovulation (De Mones *et al*., 1989; Penney and Moffitt, 2014a) suggest that secondary oocyte growth (and hence a decision to initiate recrudescence) begins prior to ovulation of the mature oocyte cohort.

Elevations in TG level in reproductive fish in this study at time points after 10 weeks post-spawning, as well as the decrease immediately before spawning, are consistent with both greater feeding and mobilization of lipid reserves during vitellogenesis. The major fates of circulating TGs are expected to be storage in muscle, mesenteric and liver lipid depots (Sheridan, 1994) and incorporation into the developing ovary during exogenous vitellogenesis (Norberg and Haux, 1985). This is supported by studies that have observed increases in plasma TG during exogenous vitellogenesis in reconditioned repeat spawning Atlantic salmon (Johnston *et al*., 1987) and prior to peak vitellogenesis in rainbow trout (Bon *et al*., 1997). In female brown trout (*Salmo trutta*), plasma TG levels decreased ~50% after spawning (Gauthey *et al*., 2015), similar to the post-spawning decrease found in non-reproductive fish in the present study. In non-reproductive fish, TG levels increased to 100–300 mg dL^−1^ by Week 30, remained in this range through the following winter, then increased to 200–300 mg dL^−1^ by the spring for skip spawners (now reproductively active, 1 year + 10–30 weeks after first spawning), remaining in this range throughout the summer. This suggests that plasma TG levels are maintained by homeostatic processes in actively feeding post-spawning female steelhead trout and that increased levels reflect both seasonality and reproductive status.

ML levels and K increased more rapidly in reproductive than in non-reproductive fish diverging at 20 weeks following the increase in TG levels. The greater ML levels and K in reproductive fish in the year following first spawning is consistent with reconditioned wild female steelhead trout ~6 months after spawning (Pierce *et al*., 2017) and fully fed versus feed restricted adult female rainbow trout over the first 20 weeks after spawning (Caldwell *et al*., 2013). Similarly, K was greater in reproductive reconditioned repeat spawning female Atlantic salmon from 18 to 34 weeks after spawning (Johnston *et al*., 1987). As spawning approached for reproductive fish, ML levels decreased and ultimately became significantly lower than ML levels in non-reproductive fish at Week 50. This likely reflects mobilization of MLs for incorporation into the ovary and reduced appetite.

K also decreased in reproductive fish over the period immediately before spawning, but this decrease was not nearly as dramatic as in plasma TG and ML levels. This was likely due to the presence of fully developed ovaries in the body cavity of the reproductive fish. The much higher K levels at second versus first spawning in reproductive fish is both because the mass of the eggs was not included in somatic mass at first spawning and because feeding and somatic growth continued through the fall prior to spawning in reproductive fish, unlike in first-time spawners. Similar results were observed in consecutive spawning reconditioned Atlantic salmon kelts; these fish experienced a minor decline in K following spawning but remained well above first spawning levels (Johnston *et al*., 1987).

At first spawning, no biologically significant differences in lipid reserve metrics were detected between subsequently reproductive and non-reproductive fish, providing no evidence for a determinative role of lipid reserves at spawning in the decision to initiate recrudescence. This does not necessarily imply that the critical period hypothesis of salmonid maturation does not hold for reproductive decisions in repeat spawners. The critical period during which reproductive decisions are sensitive to energy reserves may simply occur earlier, before first spawning for consecutive spawners, or greater than 1 year prior to second spawning. However, it is also possible that the lipid reserve metrics employed in this study did not capture the relevant physiological signals. Signalling factors associated with energy reserves, rather than energy reserves themselves, presumably directly interact with neuroendocrine mechanisms underlying this decision (Wootton and Smith, 2015). Future study of signalling factors associated with energy reserves and growth is required to elucidate mechanisms underlying reproductive decisions in salmonids.

#### Year prior to second spawning

Over the year prior to repeat spawning, TG levels were lower in consecutive spawners versus reproductive skip spawners at Weeks 0 and 10, which can be attributed to the costs of fasting, migration, spawning and recovery experienced by consecutive spawners. A comparable effect was seen in juvenile rainbow trout, in which plasma TG levels decreased during fasting and this decrease was exacerbated by swimming (Simpkins *et al*., 2003).

Over the summer before repeat spawning, ML levels continued to increase in reproductive skip spawners and thus remained much higher than those in consecutive spawners, indicating greater energy reserves in the reproductive skip spawners were due to the much longer time for recovery from first spawning. K increased in both consecutive spawners and reproductive skip spawners. Although the increase was steeper in consecutive spawners, this was not sufficient to surpass the much higher K levels in reproductive skip spawners. As K is a measure of body shape, this likely reflects increased size and greater energy stores in a variety of tissues and organs in the skip spawners, including muscle tissue, visceral lipids and the developing ovaries.

### Growth

#### Year after first spawning

MSGR diverged between reproductive and non-reproductive fish during the first 10 weeks after first spawning and remained elevated in reproductive versus non-reproductive fish through the summer growing season, although these differences were not always significant. Growth in length was generally similar to that in mass over the summer growing period, aside from the negative length growth over the first 10 weeks. Reproductive fish gained mass, whereas non-reproductive fish lost mass over the first 10 weeks after spawning. As discussed above, this is almost certainly due to differential food intake and assimilation in reproductive versus non-reproductive fish. These results are consistent with the greater spring to fall growth found in reproductive versus non-reproductive reconditioned wild Yakima River female steelhead trout kelts (Pierce *et al*., 2017) and with post-spawning mass gain in reproductive adult female rainbow trout (Caldwell *et al*., 2013).

Growth in mass decreased compared to earlier time periods in both reproductive and non-reproductive fish as annual spawn timing approached. This is likely at least in part due to seasonal growth patterns dictated by water temperature and photoperiod (Burgner *et al*., 1992). Decreases in growth as spawn timing approached were also reported in both reproductive and non-reproductive female Atlantic salmon. Moreover, similar to what was observed in the present study, decreases were more dramatic in maturing fish (Kadri *et al*., 1996). Additionally, Stead *et al*. (1999) found a correlation between decreased growth, increased levels of plasma sex steroids and decreased food consumption during later stages of maturation in Atlantic salmon. Negative mass growth (e.g. weight loss) in reproductive fish during the 10 weeks before spawning may reflect both reduced food consumption and the energetic cost of ovarian growth.

Length decrease over the immediate 10 weeks after first spawning was observed for both reproductive and non-reproductive fish. This length decrease (~1 cm) may be due to recession of the kype, a secondary sexual characteristic consisting of elongation of the lower jaw. Although kype development is more pronounced in male salmonids, it also occurs over the period before spawning in females (Vandenberghe and Gross, 1989). Consistent with this possibility, length increase in reproductive fish exceeded that of non-reproductive fish over the 10 weeks preceding spawn timing.

#### Year prior to second spawning

MSGR was greater in reproductive skip spawners than in consecutive spawners over Weeks 0–10 during the year prior to second spawning. This difference can be attributed to the impact of prolonged fasting, migration and first spawning on consecutive spawners. The gut is atrophied in post-spawning summer-run steelhead trout, and degenerative changes are found in the liver (Penney and Moffitt, 2014a). The gut-somatic index decreases linearly over time in fasted juvenile rainbow trout with a loss of ~ 40% of the relative mass of the gut over 147 days of fasting (Simpkins *et al*., 2003; Zaldua and Naya, 2014). In Atlantic salmon fasted for 50 days, restoration of the gut upon refeeding required at least 1 week, during which feed intake was reduced (Krogdahl and Bakke-McKellep, 2005). The duration of fasting and energetic demands of migration and ovarian development were substantially greater in the steelhead trout used in the present study than in the Atlantic salmon refeeding study, as indicated by a proportional lipid depletion of 93–98% observed from upstream to post-spawn migration (Penney and Moffitt, 2014b) and less than 1% wet ML mass at spawning (this study). Thus, restoration of digestive function, feeding motivation and feed intake would be expected to take at least several weeks in post-spawning female steelhead trout. LSGR was also greater in reproductive skip spawners than in consecutive spawners over Weeks 0–10 during the year before repeat spawning, consistent with post-spawning kype reduction in consecutive spawners discussed above, as well as with the impact of fasting, migration and spawning on growth on consecutive spawners.

### Estradiol-17β

#### Year after first spawning

E2 decreased following spawning regardless of reproductive status, increased to peak at 40 weeks post-spawning in reproductive fish before decreasing at second spawning and diverged between trajectories at 20 weeks after first spawning. Post-ovulatory decreases in E2 over the month after spawning have been described in female rainbow trout and Atlantic salmon (De Mones *et al*., 1989; Andersson *et al*., 2013; Caldwell *et al*., 2014). This post-ovulatory decrease may be physiologically significant in that gonadal steroids and other gonadal factors suppress plasma follicle-stimulating hormone (FSH) levels in post-ovulatory rainbow trout (Breton *et al*., 1998; Chyb *et al*., 1999). Thus, it is possible that clearance of these factors may be necessary before FSH stimulation of ovarian development can occur. The decrease in E2 late in oogenesis is consistent with previous studies in salmonids (Fostier *et al*., 1978; Whitehead *et al*., 1983; Nagler *et al*., 2012; Andersson *et al*., 2013) and likely reflects a steroidogenic shift from E2 to the maturation inducing steroid 17*α*, 20*β* dihydroxyprogesterone induced by luteinizing hormone (Nagahama, 1994; Bobe *et al*., 2006). The divergence of E2 levels 20 weeks after first spawning was due to a significantly different increase from 10 weeks in individual consecutive spawners versus a decrease in individual non-reproductive skip spawners. This indicates that the decision to engage in reproductive activity was made prior to this time point. At ~5 months after spawning, this was somewhat slower than that observed in reconditioned female wild-origin Yakima River steelhead trout (~3 months) (Pierce *et al*., 2017) and slower than in feed-restricted versus fully fed post-spawning female rainbow trout (10 weeks) (Caldwell *et al*., 2014). The differences in divergence timing may be due to fish origin and variation in metabolic rate. DNFH hatchery-origin steelhead trout are larger than Yakima River steelhead trout and much larger than rainbow trout and were held in colder water than in either of the previous studies. Both the size and temperature differences would be expected to result in a lower metabolic rate in the DNFH fish. The time between natural spawning and collection for reconditioning, as well as potential differences between natural and artificial spawning, could result in more rapid development in the Yakima River fish.

Premature decreases in E2, which would indicate arrested reproductive development after the start of exogenous vitellogenesis, were not observed in this study. Similarly, low E2 levels used to categorize non-reproductive status, as was done in the present study, coincided with low vitellogenin in skipping steelhead trout (Pierce *et al*., 2017) and low gonadosomatic index (GSI) tracked over time in skipping rainbow trout (Caldwell *et al*., 2013). Additionally, vitellogenin levels were elevated by early summer in reproductive post-spawning summer-run steelhead trout (Pierce *et al*., 2017), indicating that exogenous vitellogenesis occurs roughly during the time at which summer-run steelhead must leave ocean feeding areas to begin their spawning migration (Burgner *et al*., 1992). Thus, skipping after this time would involve a seemingly maladaptive migration pattern. Finally, salmonids are thought to commit to a reproductive cycle when oocytes begin to accumulate cortical alveoli, further narrowing the transition to that of the beginning of secondary oocyte development (Campbell *et al*., 2006; Taranger *et al*., 2010). Taken together with the present results, we interpret these findings as suggesting that post-spawning summer-run steelhead likely halt reproductive development in the perinucleolar stage of oocyte development, coinciding with the ‘spent-recovery’ stage of ovarian development, representing the ‘resting’ form of skipped spawning as defined by Rideout and Tomkiewicz (2011).

#### Year prior to second spawning

E2 was low through the winter in non-reproductive fish but increased in Year 2 from Week 0 (+1 year) to Week 10 to ~10-fold higher in skip spawners than consecutive spawners and comparable to levels in consecutive spawners at Week 20. This indicates that the decision to initiate reproductive activity occurred by Week 10 in skip spawners and suggests that reproductive development was accelerated in reproductive skip spawners versus consecutive spawners. Consistent with this idea, spawning was later in consecutive versus skip spawning steelhead trout (Jenkins *et al*., 2018), as in consecutive versus first-time spawning Atlantic salmon (Pankhurst *et al*., 2011). Plasma E2 levels remained greater in reproductive skip spawners versus consecutive spawners as oogenesis proceeded through the summer growing season and levels increased. Similarly, plasma E2 levels were lower in consecutive spawning Atlantic salmon versus first-time spawners at sampling time points ~10–20 weeks after spawning (Pankhurst *et al*., 2011). The higher E2 levels in reproductive skip spawners versus consecutive spawners may have resulted in the 14% greater size-adjusted total egg mass found in these fish at the time of second spawning (Jenkins *et al*., 2018). Both the delay in initiation of maturation in consecutive spawners and the greater reproductive investment observed in skip spawners at second spawning can be attributed to the effects of recovery from first-time spawning on consecutive spawners. These effects were likely largely mediated by energetic status, as discussed above. However, in addition, reproductive development in consecutive spawners may have been directly affected by recovery from first spawning due to time required for clearance of steroids and other gonadal factors, continued steroid production by post-ovulatory follicles and tissue resorption and remodelling of the post-ovulatory ovary (De Mones *et al*., 1989; Chyb *et al*., 1999; Caldwell *et al*., 2014). The impact of first spawning on reproductive development in consecutive spawners illustrates the benefits of having time to recover from first spawning that occur before the early stages of oogenesis.

## Conclusions

By 10 weeks after first spawning, growth rate and TG levels were greater in reproductive than in non-reproductive fish. This suggests that the decision to initiate ovarian recrudescence takes place by 10 weeks after spawning in consecutive spawners. During the year prior to second spawning, plasma E2 levels increased by 10 weeks in reproductively active skip spawners, implying that the decision to engage in reproductive activity occurs by 10 weeks + 1 year after spawning in skip spawners. The increase in plasma E2 was delayed by 10 weeks in consecutive spawners compared to reproductive skip spawners, suggesting that reproductive development was delayed due to the effects of first spawning. After first spawning, reproductive fish recovered more quickly than non-reproductive fish, consistent with stimulation of feeding by initiation of ovarian recrudescence. Furthermore, while consecutive spawners sustained greater growth rates during oogenesis, skip spawners accumulated higher levels of energy reserves and had higher E2 levels, which may be causally related to the 14% greater reproductive investment found in skip spawners at the time of second spawning (Jenkins *et al*., 2018). Further studies using this experimental system should provide additional insights into consecutive and skip spawning biology, as well as directly informing the management of steelhead kelt reconditioning programs.

## Supplementary Material

Supplementary_Figure_1_coz038
